# High Tensile Ductility and Strength in Dual-phase Bimodal Steel through Stationary Friction Stir Processing

**DOI:** 10.1038/s41598-019-38707-3

**Published:** 2019-02-13

**Authors:** H. S. Arora, A. Ayyagari, J. Saini, K. Selvam, S. Riyadh, M. Pole, H. S. Grewal, S. Mukherjee

**Affiliations:** 1 Surface Science and Tribology Lab, Department of Mechanical Engineering, Shiv Nadar University, Uttar Pradesh, 201314 India; 2 Department of Materials Science and Engineering, University of North Texas, Denton, Texas 76203 USA; 3 Center for Nanoscale Materials, Argonne National Laboratory, Argonne, IL 60439 USA

## Abstract

The combination of high strength and good ductility are very desirable for advanced structural and functional applications. However, measures to enhance strength typically lead to ductility reduction due to their inverse correlation, nano-grained structures for an instance. Bi-modal grain structure is promising in this regard, but its realization is limited by multiple complex processing steps. Here, we demonstrate a facile single-step processing route for the development of bimodal grain structure in austenitic stainless steel, SS316L. The bimodal structure comprised of fine martensite grains (<500 nm) sandwiched between coarse austenite grains (~10 µm). The dual-phase bimodal structure demonstrated higher yield strength (~620 MPa) compared to ultra-fine grain structure (~450 MPa) concurrent with high uniform tensile ductility (~35%). These exceptional properties are attributed to unique dual-phase, bimodal grain structure which delayed the onset of plastic instability resulting in higher strength as well as larger uniform elongation and work-hardening rate. Our approach may be easily extended to a wide range of material systems to engineer superior performance.

## Introduction

The trade-off between strength and ductility remains one of the major bottlenecks in the development of advanced structural materials. Attempts to enhance strength invariably results in ductility reduction through restricted dislocation movement. Heterogeneities at microstructural length-scales through hierarchal architecture have been utilized for overcoming strength-ductility paradox including, nano-twinned grains^1,2^, heterogeneous lamellae^3–5^, laminate^6^, harmonic^6,7^ and bimodal structures^8–10^. Bimodal microstructure, comprising of fine grains in the matrix of coarse grains or vice-versa are promising in this regard. Finer grains provide high strength while the coarser grains contribute to appreciable ductility through sufficient dislocation accommodation. However, coarser grains in bimodal structure leads to lower strength compared to ultrafine-grained (UFG) microstructure for the same alloy. Microstructure design wherein the loss in strength from coarse grains can be compensated, is likely to result in superior mechanical properties, even better than conventional ultrafine and bimodal grain structures^11,12^.

The realization of bimodal grain structure is limited by multiple complex processing steps involving a combination of severe plastic deformation, heat treatment and/or cryogenic processing^8,13–16^. Achieving such desirable microstructures is even more challenging in high strength materials such as stainless steels. This is attributed to their high resistance to plastic deformation and limited sensitivity to heat-treatment. In addition, bimodal grain structure obtained in all previous studies demonstrated lower strength compared to the corresponding ultra-fine grain structure. Here, we demonstrate a novel, single-step processing route for the development of dual-phase, bimodal grain structure in stainless steel. In contrast to previous studies, the bimodal structure in the current study showed higher strength and work-hardening rate compared to the ultra-fine grain structure without compromising tensile ductility. The reduction in strength from coarse grains is more than compensated by fine martensite grains distributed in the austenite matrix.

## Materials and Methods

The material used in the current investigation was austenitic stainless steel, 316 L. Submerged friction stir processing (FSP) was performed to tailor the surface properties using a pin-less tool made of tungsten carbide with 12 mm shoulder diameter. FSP was performed with the following processing parameters: (1) rotational speed of 1800 rpm and 0.4 mm plunge depth, tool was traversed along the longitudinal direction at 20 mm/min on the workpiece while submerged in a pool of liquid (mixture of distilled water and ethanol in equal proportion) at 0 °C; (2) tool was rotated at 1800 rpm at a particular location of the workpiece for a period of 15 minutes while submerged in a pool of liquid, resulting in localized straining. A special purpose FSP fixture was fabricated for holding the sample while submerged in the liquid bath. The FSP fixture was connected to an external chiller through inlet and outlet ports for constant flow of coolant at nearly 100 ml/min. All samples were polished down to 3000 grit followed by electro-polishing in 10% oxalic acid solution at 650 mV for 2 minutes. The grain size and phase distribution for the processed and unprocessed samples were obtained using electron back scatter diffraction (EBSD) and X-ray diffraction. EBSD analysis was conducted using FEI Quanta 3D FEG using step size of 0.1 µm. Grain size distribution and statistical deviation for all specimen was obtained from EBSD image analysis. Specimen for transmission electron microscope (TEM) studies were prepared using FEI Nova NanoLab 200^TM^ focused ion beam (FIB). Microstructure observation was carried out on FEI Tecnai F20 field emission gun TEM operating at 200 kV. For tensile testing, dog-bone shaped mini-tensile specimens with dimensions of 5 mm × 1.25 mm × 0.4 mm were prepared using computer numerical control (CNC) machine. All tests were done at room temperature at a strain rate of 10^−3^ s^−1^. Each sample was tested two times to ensure repeatability of results.

## Results and Discussion

**Figure 1:**
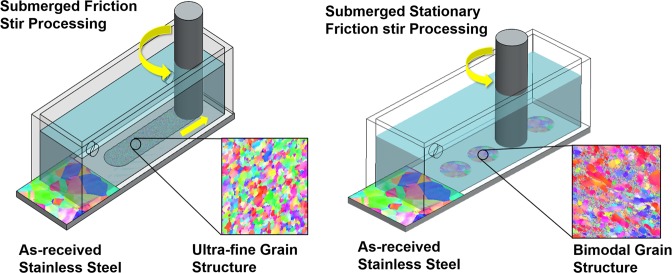
(**a**) Schematic showing the submerged friction stir processing (SFSP) and submerged stationary friction stir processing (SSFSP) techniques used in the current study. The rotating tool traverses along the workpiece during SFSP wheras, the tool rotates at a particular location of the workpiece during SSFSP. The workpiece remained submerged in a pool of low temperature liquid in both the cases. While SFSP resulted in ultra-fine grain structure, SSFSP produced dual-phase bimodal grain structure.

**Figure 2:**
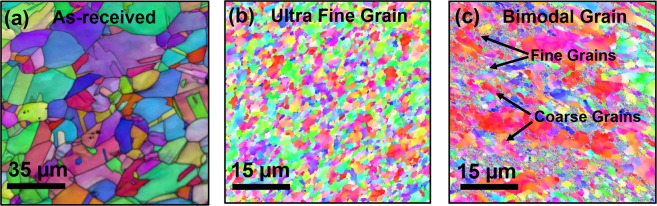
Electron back scatter diffraction images for (**a**) as-received stainless steel, SS316L, (**b**) ultra-fine grain structure in SS316L, and (**c**) bimodal grain structure in SS316L. The as-received steel had an average grain size of 22 µm which got refined to 0.9 µm for the ultra-fine grain structure. The bimodal steel showed fine martensite grains (<500 nm) embedded in the matrix of coarse austenite grains. The average grain size for the bimodal grain structure was found to be 3.5 µm.

**Figure 3:**
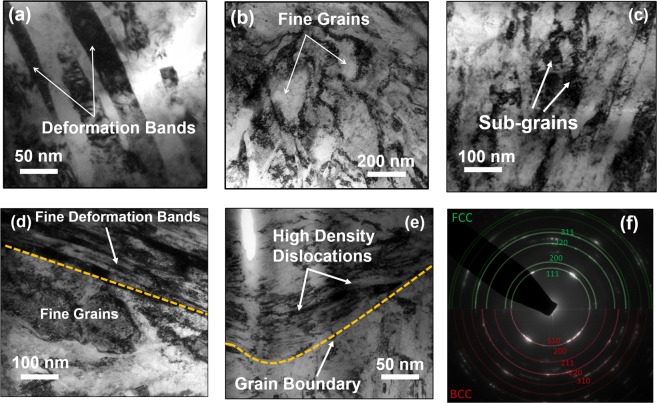
Transmission electron microscope (TEM) images for (**a**) ultra-fine grain steel; bimodal steel showing (**b**) finer grains of the order of 200–400 nm, (**c**) fine sub grains, (**d**) fine deformation bands, (**e**) high density dislocation along the grain boundary, and (**f**) selected electron diffraction pattern (SADP). The UFG steel showed elongated deformation bands with high density dislocation. SADP for bimodal specimen showed both FCC and BCC phases. The BCC phase corresponds to martensite.

Schematic representations of the processing routes used in this study are summarized in Fig. 1. The processing involves plunging a rotating cylindrical tool and traversing it along the length of the specimen, resulting in ultra-fine grain structure. In contrast, rotating the tool at a specific location on the workpiece resulted in bimodal grain structure comprising of fine grains in a matrix of coarse grains. The work-piece remained immersed in a low temperature liquid during processing in both the cases. The depth of the processed region in both the cases was found to be nearly 300 µm. The electron back scatter diffraction images for the as-received, ultra-fine and bimodal grain structure are shown in Fig. 2a–c. The as-received steel showed a wide variation in grain size with average value of 22 µm and standard deviation of 8.5 µm. The UFG steel had significantly refined microstructure with average grain size of nearly 0.9 µm and small standard deviation of about 0.33. In contrast, the bimodal grain steel showed extremely fine grains (<500 nm) embedded in the matrix of coarse grains of nearly 10 µm in size. The average grain size of bimodal specimen was nearly 3.5 µm. The finer grains in the bimodal steel were martensite while coarse grains were austenite as shown by EBSD phase map (Fig. S1). The volume fraction of martensite was nearly 30% and 8% in bimodal and UFG specimen respectively, while as-received steel had predominantly austenite. X-ray diffraction (XRD) supported the presence of martensite phase in the bimodal specimen (Fig. S1). XRD for UFG steel showed austenite as the primary phase, but there was no indication of the presence of martensite likely due to the small volume fraction. TEM analysis of the ultra-fine grain steel, shown in Fig. 3a, revealed a microstructure with large volume fraction of elongated deformation bands divided by thin boundaries. Within the deformation bands, high density of dislocations can be observed. The average band width is roughly 40–50 nm. Figure 3b,c shows fine grains of the order of 200–400 nm and sub-grains in the bimodal specimen. The bimodal specimen also showed extremely fine deformation bands and sub-grain features consisting of regions with very high and low dislocations density (Fig. 3d). This heterogeneity divided the microstructure into fine/coarse grains structure, which agrees well with the EBSD results. Figure 3e shows the pile up of dislocations along the grain boundaries. The selected area diffraction pattern (SADP) for the bimodal specimen showed BCC martensite in FCC austenite matrix (Fig. 3f) in line with the EBSD phase analysis.

**Figure 4:**
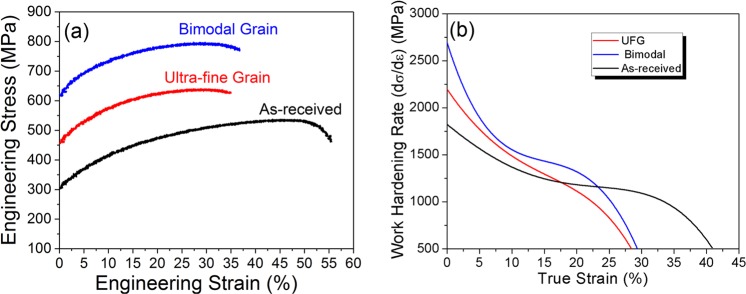
Engineering stress-strain curve for as-received stainless steel, ultra-fine grain steel and bimodal grain steel; (**b**) Work-hardening rate as a function of strain for all the investigated specimen. The bimodal structure showed higher yield strength of 620 MPa compared to ultra-fine grain structure (~450 MPa) concurrent with high uniform tensile ductility (~35%).

**Figure 5:**
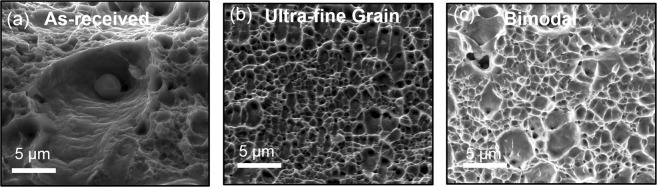
Fractographs showing the fracture surfaces from tensile testing for (**a**) as-received steel, (**b**) ultra-fine grain steel, (**c**) bimodal steel. All samples show similar features of extensive dimple formation indicating appreciable plastic deformation prior to the failure.

The engineering stress-strain curve for all specimen are shown in Fig. 4a. The as-received steel showed yield strength of 300 MPa and 50% uniform elongation. As expected, the yield strength of UFG steel showed an increase to 450 MPa, concurrent with decrease in uniform elongation to 30%. The bimodal specimen demonstrated highest yield strength of 620 MPa with elongation of 35%. This is in sharp contrast to previous reports that showed lower yield strength of bimodal specimen over UFG/nano-grained specimen^17–20^. Also, the bimodal steel showed highest rate of work-hardening followed by UFG and the as-received steel (Fig. 4b). The variation of work-hardening rate as a function of true strain was however similar for all three specimens with three distinct stages. An initial high rate of work hardening was followed by reduced near steady-state stage with minimum work-hardening rate towards higher strain values. The fractographs of all tensile tested specimen showed similar features of extensive dimple formation (Fig. 5), indicating appreciable plastic deformation prior to the failure.

Grain refinement by traversing the rotating tool over workpiece is well known for friction stir processing^21–24^. It proceeds by primary recrystallization whereby the cold-worked structure is replaced by the recrystallized grains. The recovery process in low stacking fault energy materials, such as austenitic stainless steel, is relatively slow and undergoes dynamic recrystallization when critical deformation conditions are met. Typically, the recrystallization phenomena in these materials involve generation of new grain structure by distinct nucleation and growth phases, commonly referred as discontinuous dynamic recrystallization (DDRX)^25^. During deformation, prior grain boundaries and high angle grain boundaries acts as nucleation sites for the formation of new grains. As the recrystallization proceeds, the nucleated grains form a thickening band of recrystallized grains in a necklace-type pattern which eventually leads to a fully recrystallized structure. Mathematically, the condition for DDRX to occur is given by the relation: ${{\rho }^{3}}_{m}/\mathop{\varepsilon }\limits^{.} > 2{\gamma }_{b}/KMLG{b}^{5}$ where, ${\rho }_{m}$ is the mobile dislocation density, $\mathop{\varepsilon }\limits^{.}$ is the strain rate, ${\gamma }_{b}$ is the grain boundary energy, *K* is a constant fraction of the dislocation line energy that is stored in the newly formed grains, *L* is mean slip distance of dislocations in these grains, *M* is the boundary mobility, *G* is the shear modulus, and *b* is the Burger’s vector. The inequality suggests that higher dislocation density favours DDRX. The energy stored in the structure during deformation is the driving force for the completion of recrystallization^25^. The origin of bi-modal structure is likely related to the mechanism of microstructural evolution during DDRX. The application of external strain causes sliding/migration of the parent grain boundaries. The migrating grain boundary acts as a nucleation site for the development of new stress-free grains, the dimeter (*d*) of which can be expressed by the relation: $d=MLG{b}^{3}{\rho }_{m}^{2}/\mathop{\varepsilon }\limits^{.}$^25^ where *M*, *L*, *G*, *b*,${\rho }_{m}$ and $\mathop{\varepsilon }\limits^{.}$ have ususal meanings as above. Thus, higher the strain-rate, lower is the nucleated grain size. As the grain boundaries sweep through the matrix during recrystallization and grain growth, the dislocation density decreases in the migrating regions, whereas their density inside the recrystallized grain tends to increase due to concurrent deformation. The growth rate of nucleated grains during DDRX is dependent on the dislocation density. Typically, the grain growth rate reduces with increase in dislocation density and eventually, the nucleated grains ceases to grow. Nucleation of new grains at migrating grain boundaries may also limit the growth of nucleated grains during DDRX. Since, the strain rate during friction stir processing is proportional to tool rotational speed^26^, the nucleated grains are likely to be very fine due to high rotational speed used in the current study. During stationary processing, the material was strained for a significantly longer time compared to conventional FSP. The dislocation density in the nucleated grains is much higher due to aforementioned high localized staining (as evidenced by TEM results), which consequently limits their growth and the expansion of necklace pattern into the parent grain. This may explain the observation of complete recrystallized parent grains in juxtaposition with fine grain structure resulting in the observed bimodal grain structure. To validate this hypothesis, stationary processing was performed for a lower time of 5 minutes. With decrease in processing time, the bimodal grain structure became coarser (Fig. S2(a)). The average size of austenite grains increased to nearly 15 µm while fraction of fine martensite grains got reduced compared to 15 minutes stationary processing (Fig. S2(b)). The dislocation density for lower processing time is likely to decrease resulting in larger growth of nucleated grains and thus coarser bimodal grain structure. In contrast, nucleation of martensite phase through recrystallization is highly unlikely as it is a diffusion-less transformation. During stationary processing, the material is strained locally resulting in pile up of dislocations along the grain boundaries (Fig. 3e). The stacked up dislocations form slip bands with bundles of twins and faults which act as martensite nucleation sites^27^. In addition, an increase in inelastic strain from deformation causes nano-twins and sub-grain features (Fig. 3c) with higher stacking faults that can transform into martensite after a critical strain. The critical strain energy for austenite to martensite transformation is proportional to the difference in their Gibbs free energy (ΔG^Ɣ→ά^). Deformation induced stress acts as an additional driving force for martensite transformation by increasing the internal strain energy. Accumulation of internal energy through dislocations is limited by the recovery process^28,29^ and stabilized austenite phase, thus constraining continual growth of martensite phase. This resulted in the unique dual-phase bimodal grain structure.

The bimodal specimen demonstrated higher work hardening, higher yield strength as well as larger elongation compared to UFG specimen. Work hardening in the material delays the onset of tensile instability which is given by Conside’re criteria^30,31^: ${(\partial \sigma /\partial \varepsilon )}_{\mathop{\varepsilon }\limits^{.}}\le \sigma$, where $\sigma$ and $\varepsilon$ are the true stress and strain respectively. Typically, fine grain materials show plastic instability (i.e. loss of work hardening) at lower strains. This is attributed to lower dislocation density in fine grains. Higher strength and ductility seen for the bimodal specimen implies delayed plastic instability through appreciable strain-hardening. The unusual properties of bimodal specimen are attributed to its unique dual-phase microstructure. The fine martensite grains embedded in coarse austenite matrix of bimodal steel may be modelled as a dispersion strengthened system^32,33^. For dispersion strengthened alloys, the work-hardening rate primarily depends on the average dislocation density around particles interacting with primary dislocations^32^. Mathematically, work hardening rate for dual-phase alloys is given as: $d\sigma /d\varepsilon =\alpha \mu {(f/d)}^{1/2}{(b/\varepsilon )}^{1/2}$ where, $\sigma$ is the true stress, $\varepsilon$ is the true strain, *α* is a constant, *μ* is shear stress, *f* is the volume fraction of second phase, *d* is the particle size and *b* is the burgers vector. Linear dependence of work hardening rate on ${(f/d)}^{1/2}$ implies that increasing the volume fraction of hard second-phase at constant diameter increases the work-hardening rate as well as the tensile strength with concurrent decreases in elongation. In contrast, decreasing the diameter of second phase at constant volume fraction increases the work-hardening rate. High dislocation density around hard, non-deformable fine second phase favors appreciable elongation before failure. Thus, strength can be improved by increasing the volume fraction of second-phase while work-hardening and ductility can be enhanced by reducing the diameter of second-phase. The average size/diameter of martensite grains in bimodal specimen (<500 nm) was of the same order or smaller compared to the average grain size of the UFG specimen (~0.9 µm). The martensite volume fraction in bimodal specimen was considerably larger (~30%) compared to UFG steel (~8%). Therefore, simultaneous higher yield strength and work-hardening rate for bimodal specimen is attributed to higher volume fraction of finer martensite grains. The larger elongation for bimodal specimen may be explained based on high dislocation density at hard non-deformable martensite grains and high dislocation carrying capacity of coarse austenite grains.

In summary, we demonstrated a novel processing pathway for developing unique dual-phase bimodal grain structure comprising of fine martensite grains embedded in the matrix of coarse austenite grains. The bimodal grain structure showed higher yield strength of 620 MPa compared to 450 MPa for the ultra-fine grain structure while maintaining high tensile ductility. The unusual properties of bimodal specimen are attributed to its unique dual-phase microstructure. The large fraction of fine martensite grains in the coarse austenitic phase delayed the onset of plastic instability resulting in significant work-hardening for bimodal grain structure, providing a pathway for circumventing the strength-ductility paradox in structural alloys.

## Supplementary information

Supporting Information

## Data Availability

All data generated or analyzed during this study are included in this paper. Raw datasets are available from the corresponding author, upon receipt of a reasonable request.
