# Supplementary material for: High Tensile Ductility and Strength in Dual-phase Bimodal Steel through Stationary Friction Stir Processing
Source: Sci Rep. 2019 Feb 13;9:1972. doi: 10.1038/s41598-019-38707-3 (PMC6374438; doi:10.1038/s41598-019-38707-3)
Supplement: Supplementary file 1 — Supporting Information [file 41598_2019_38707_MOESM1_ESM.doc]

**Supporting Information**

High Tensile Ductility and Strength in Dual-phase Bimodal Steel through Stationary Friction Stir Processing

H.S. Arora ^#, 1^, A. Ayyagari^2,3^, J. Saini^1^, K. Selvam^1^, S. Riyadh^2^, M. Pole^2^, H.S. Grewal^1^, S. Mukherjee^,*, 2^

^1^Surface Science and Tribology Lab, Department of Mechanical Engineering, Shiv Nadar University, Uttar Pradesh, India 201314

*^2^Department of Materials Science and Engineering, University of North Texas, Denton, Texas 76203, USA*

*^3^Center for Nanoscale Materials, Argonne National Laboratory, Argonne, IL, 60439 USA*

*^#^E-mail:* [*harpreet.arora@snu.edu.in*](mailto:harpreet.arora@snu.edu.in)

*^*^Email:* [*Sundeep.Mukherjee@unt.edu*](mailto:Sundeep.Mukherjee@unt.edu)


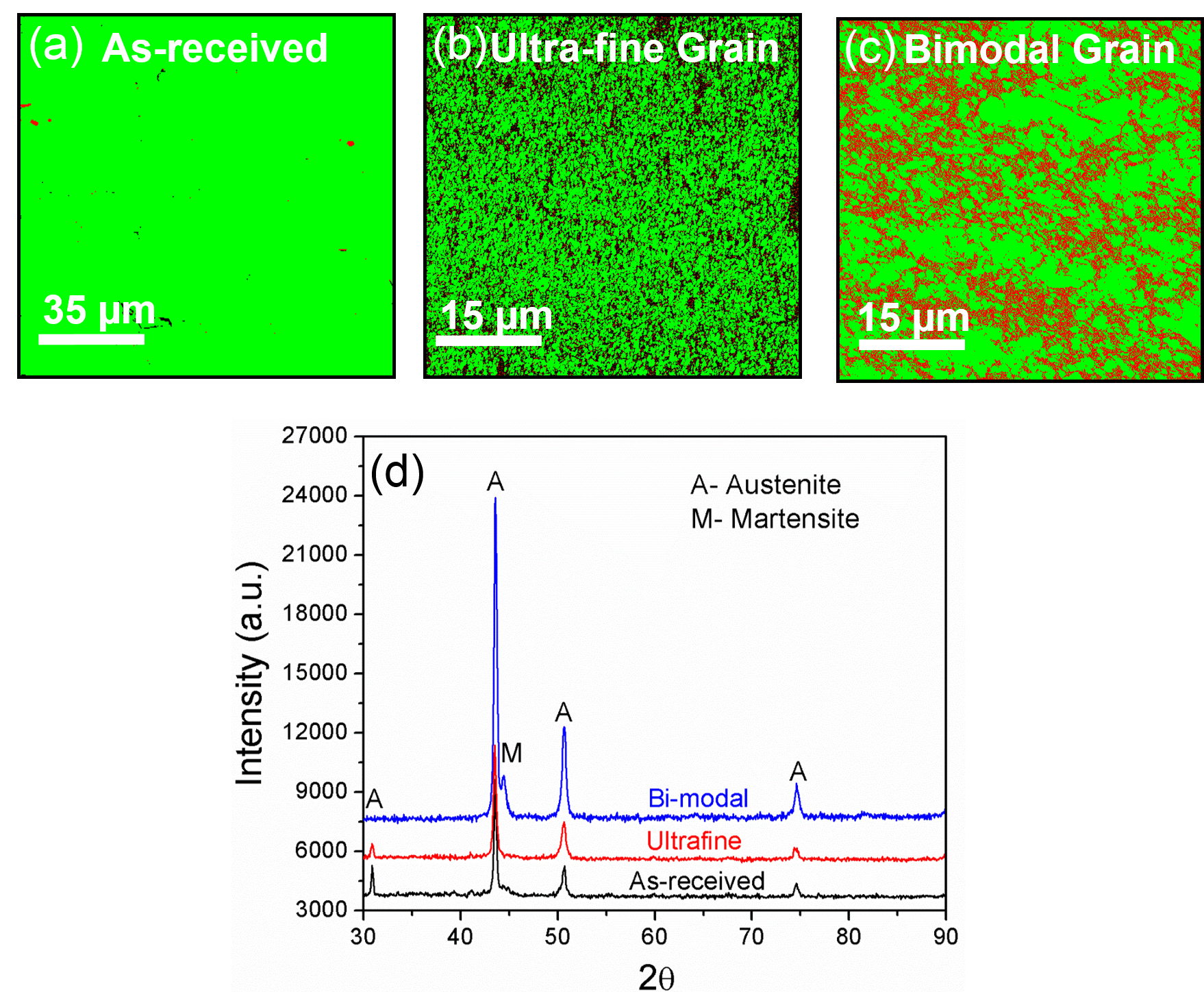


**Figure S1**: Electron back scatter diffraction (EBSD) phase map for (a) as-received stainless steel, (b) ultra-fine grain steel, (c) bimodal grain steel; (d) X-ray diffraction (XRD) analysis for all the three specimen. EBSD phase map indicate austenite phase in as-received steel, austenite and martensite for both ultra-fine grain and bimodal grain steel. XRD results did not show any evidence of martensite in the ultrafine grain steel due to its small fraction. Green color in the phase map indicate austenite phase while red color indicates martensite.


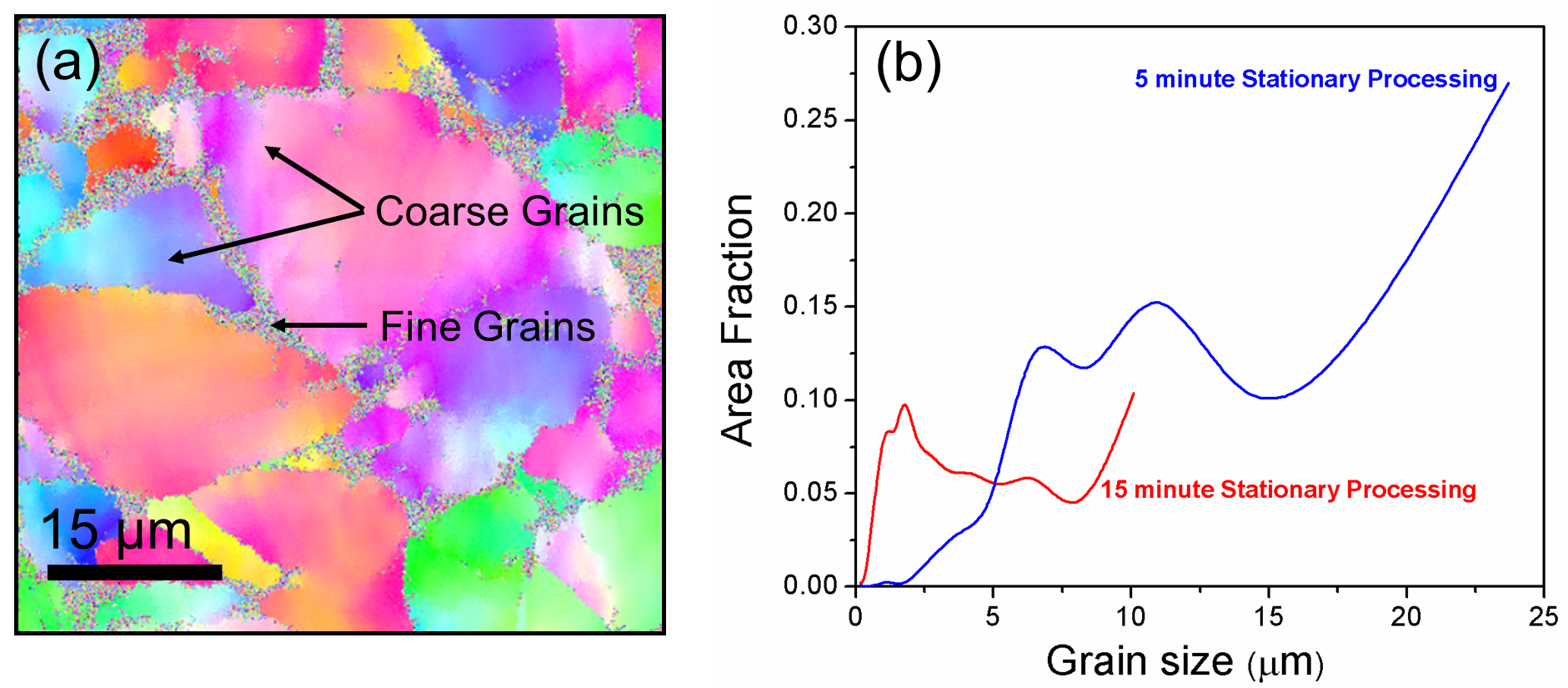


**Figure S2:** Electron back scatter diffraction (EBSD) phase map for stainless steel processed using stationary submerged friction stir processing for 5 minutes. The processed samples shows a bimodal grain structure comprising of nearly 15 µm austenite grains with fine martensite grains; (b) Comparison of grain size distribution for 5 minute and 15 minute stationary processing. The fraction of fine grains is larger for 15 minute processing.
